# Temporal and Spatial Changes in the Material Exchange Function of Coastal Intertidal Wetland—A Case Study of Yancheng Intertidal Wetland

**DOI:** 10.3390/ijerph19159419

**Published:** 2022-08-01

**Authors:** Lingjun Dai, Hongyu Liu, Yufeng Li

**Affiliations:** 1 School of Geography, Nanjing Normal University, Nanjing 210023, China; 161301031@njnu.edu.cn; 2 School of Marine Science and Engineering, Nanjing Normal University, Nanjing 210023, China; pandalee_0826@163.com

**Keywords:** material exchange function, intertidal wetland, *S. alterniflora*, temporal and spatial changes, Yancheng

## Abstract

As a transition zone between the terrestrial ecosystem and the marine ecosystem, coastal intertidal wetland provides an important place for material circulation and energy exchange, and it is nature’s most precious resource. The ecological health of intertidal wetlands is an important prerequisite for sustainable green development. The material exchange function objectively and accurately reflects the material balance and ecological health of intertidal wetlands in the coastal zone. This paper uses remote sensing, geographic information technology, and model methods to objectively and accurately assess the temporal and spatial changes in the material exchange function of intertidal wetlands, providing a feasible method for studying the material exchange function of coastal wetlands. The material exchange capacity of wetlands in intertidal zones is affected by many factors, such as vegetation, topography, and base soil. After the invasion of the alien species *Spartina alterniflora Lois.*, the elevation of the *Suaeda salsa* beach increased by 0.3 m. The elevation of the *Phragmites australis* beach rose by 0.14 m. The average elevation of *S. alterniflora* increased by 1.24 m. The material exchange capacity of the intertidal zone decreased by 25%. The functioning of the material exchange between sea and land changed significantly, and the area with a high level of material exchange function capacity was reduced by 71%.

## 1. Introduction

Coastal intertidal wetland is the most active ecosystem and is located in the transition zone between ocean and continent [1]. It is an important channel and a place for material circulation and energy exchange between the ocean and the surrounding environment [2,3]. The material exchange function of intertidal wetland maintains the ecosystem structure, process, and functional foundations and has a profound impact on the succession and degradation of the intertidal ecosystem. For this reason, understanding and scientifically assessing the temporal and spatial changes in the material exchange function of the intertidal zone ecosystem has important practical significance. At present, studies on the material exchange function of intertidal wetland ecosystems in coastal areas are in the relatively early stage. In 1974, Odum pioneered an experiment concerning the importance of the nutrient inputs of tidal water in the intertidal zone for maintaining the high productivity of *S. alterniflora* [4]. On this basis, it was proposed that the intertidal wetland was based on dissolved organic matter. The conclusion that carbon, and particulate organic carbon especially, exports organic carbon to nearshore waters has become the basic theory used in a great deal of future research and management decisions [5,6,7]. In recent years, with the urgent need for coastal ecological protection and management, European and American researchers have successively carried out research on wetland evaluation methods, with intertidal wetland deposition as the main function; they obtained a large number of research results [8,9,10,11]. For example, there is research that uses coastal wetland hydrogeomorphological indicators to establish the material deposition model of intertidal wetland and uses the evaluation model of the nutrient and organic carbon exchange function to evaluate the material exchange function of the intertidal zone. Research on the material exchange function of intertidal wetlands started relatively late, and a series of qualitative and quantitative studies have been carried out on the mechanism of the nutrient and organic matter migration of wetlands, on the calculation of exchange flux, and on seasonal dynamic changes [12,13,14,15]; however, research rarely involves the evaluation of the material exchange function of the intertidal zone. Although some scholars assessed the material exchange capacity of wetlands by constructing a typical intertidal material exchange function index in the Bohai Bay [16], they lacked a spatial perspective to reveal changes in the material exchange function of the intertidal zone.

The definition of the material exchange function of intertidal wetlands refers to the exchange capacity between tidal flats and ocean tidal waters. Its ecological significance reveals the function of nutrients in tides in maintaining ecological health and the material balance between land and sea [17,18]. When seawater immerses the surface of the wetland in the intertidal zone, a process of material exchange and energy exchange occurs [19,20]. However, due to the differences in the spatial locations of different intertidal wetland ecosystems, there are spatial differences in the material exchange function of the intertidal zone. These spatial differences are the material basis for determining the spatial differentiation of the types of intertidal wetland ecosystems. Some of these differences are decisive factors that affect the spatial heterogeneity of the material exchange function of intertidal wetlands, such as the hydrological conditions of wetlands, including tidal flooding time, inundation frequency, and the relationship between wetland and tidal water [7,9], as well as soil properties, elevation and slope, and vegetation coverage on the wetland surface [10,21]. Wetland plant biomass was found to be driven by soil water content [22,23]. Miller used the Manning coefficient to characterize the impact of intertidal wetlands on the material exchange capacity, revealing that a large area of *S. alterniflora* can reduce wave height by 80% and weaken wave energy by 95% [24,25], seriously affecting the material exchange function of the intertidal zone. How to scientifically assess the spatial differences in the material exchange function and the temporal and spatial evolution of the intertidal wetland ecosystem in coastal areas is a question of scientific interest. The main objectives include: (1) achieving a scientific understanding of the factors that affect the spatial differences in the material exchange function of intertidal wetlands; (2) constructing a scientific evaluation method for the material exchange function of intertidal wetlands; and (3) elucidating the material exchange function of intertidal wetlands, including the characteristics and laws of the temporal and spatial evolution of the exchange function.

In this study, a typical intertidal wetland in Yancheng, Jiangsu Province, China was selected as a case study. Based on the evolution characteristics and laws of a silty intertidal wetland ecosystem and the impact of the invasion of *S. alterniflora* on the intertidal wetland ecosystem, an analysis was carried out by constructing a material exchange function index method for the intertidal wetland and comprehensively using remote sensing and GIS technology. This research work investigated the temporal and spatial changes in the material exchange function of intertidal wetlands, scientifically assessed the impact of *S. alterniflora* invasion on the material exchange function of intertidal wetlands, and provided a scientific basis for the protection and restoration of intertidal wetlands.

## 2. Study Area

**Figure 1:**
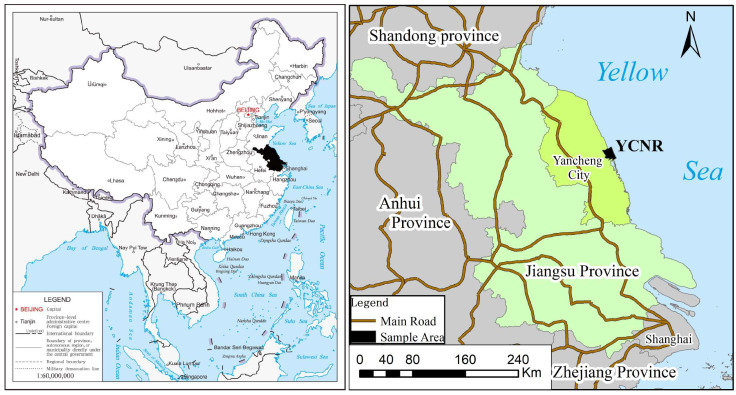
Map of the study area.

The study area is located in the coastal zone of Yancheng, Jiangsu, east of the Yellow Sea, in the area ranging from 119°27′ E to 120°54′ E and from 32°34′ N to 34°28′ N (Figure 1). The topography of the region is plain. The north and south regions were formed by the sedimentation of sediment carried by the Yangtze River and the Yellow River and through the action of ocean tides and wind waves. These regions are silty intertidal wetlands. The study area is located in a transition zone from subtropical to warm temperate. The annual sunshine time is 1956.5 h, the annual average temperature is 15.8 Celsius, and the annual precipitation is 697.3 mm. The type of wetland ecosystem found in the area is well preserved, and the impact of *S. alterniflora* invasion is prominent. Yancheng Nature Reserve, which is a Ramsar wetland of international importance, joined the Convention Concerning the Protection of the World Cultural and Natural Heritage in 2019. The unique tidal wetland ecosystem in the reserve is the habitat for rare birds, an important resting place for migratory birds from northeast Asia and Australia, and an important wintering place for waterfowl. This area is also rich in wetland biodiversity, and it occupies a very important position in international biodiversity conservation.

## 3. Methods

**Figure 2:**
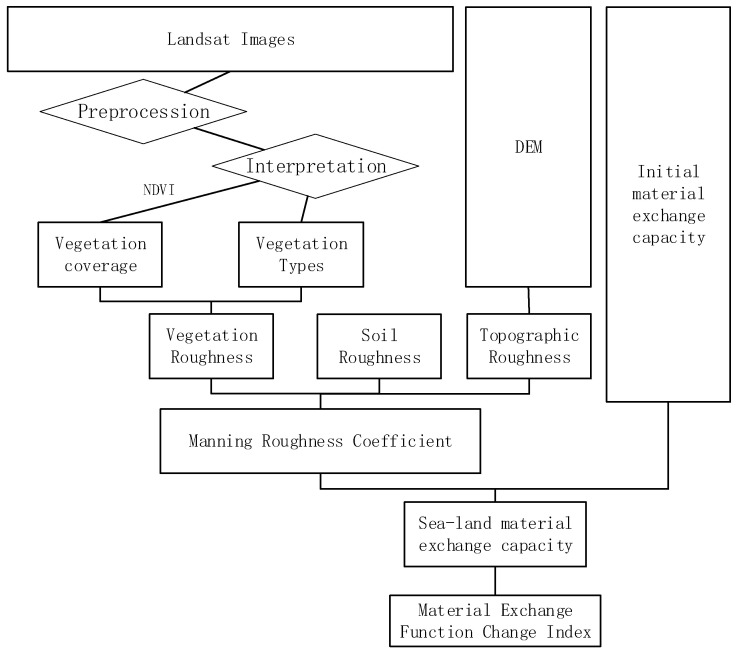
Conceptual model for evaluating the function of land–sea material exchange in the intertidal zone.

The Yancheng coastal wetland is a typical silty intertidal wetland. Affected by ocean tides and material deposition, the topography of the intertidal zone decreases from land to sea, and the ecosystem types present a zonal distribution pattern. From land to sea, *Phragmites australis (Cav.) Trin. ex Steud.*, *Suaeda salsa (Linn.) Pall.*, and *S. alterniflora* are distributed on the mudflat. Among them, *S. salsa* is the pioneer species of intertidal wetlands in the region. *S. alterniflora* was introduced to China in 1979 and was planted in Yancheng, Jiangsu in 1982. It is now listed as an invasive species. After 2000, *S. alterniflora* was distributed in belts, and its connectivity was enhanced, which had a serious impact on the adjacent *S. salsa* ecosystem. In order to scientifically reflect the temporal and spatial changes in the material exchange function of intertidal wetlands in the region, our study used topographic roughness, bare soil roughness, and vegetation roughness as factors affecting the exchange capacity between land and sea [24,25]. *S. alterniflora*, a globally distributed invasive species, exists to varying degrees in the coastal areas of China. In the 1980s, *S. alterniflora* was introduced into China due to it being considered suitable for wave breakers and dikes. Due to its strong reproductive ability, it spread rapidly. *S. alterniflora* has spread viciously in some places, affecting the local marine ecology. After *S. alterniflora* invaded coastal wetland ecosystems with its strong competitiveness, adaptability to the environment, and strong reproductive ability, it changed the structure of the vegetation community and reduced its richness and diversity; thus, it disrupted the balance of the original ecosystem [26]. The invasion of *S. alterniflora* into the community makes the total nitrogen content, nitrogen storage, and nitrogen flow stall rate in soil significantly higher than the values observed for *P. australis* and *S. salsa* [27,28]. Initially, after the invasion of *S. alterniflora*, the richness and diversity of soil bacteria and fungi in *S. salsa* and *P. australis* communities increased significantly, but with the passage of years, these levels showed a trend of first rising and then declining [29,30,31]. After the invasion of *S. alterniflora*, in addition to replacing the original plant through competition, it also hybridized with the original plant to invade the gene of the original species, causing the gene of the original plant to change [32]. By establishing the Manning roughness coefficient for the system of intertidal wetlands (Figure 2), we simulated the temporal and spatial evolution characteristics and laws of the land–sea material exchange function of coastal wetlands.

### 3.1. Acquisition and Processing of Relevant Data

#### 3.1.1. Wetland Ecosystem Types and Coverage Data

Landsat images from 1980, 1985, 1990, 1995, 2000, 2005, 2010, 2015, and 2017 were selected as the data source for our study (See Table 1). The selection criteria for remote sensing images included: (a) images were taken between August and November, when *S. alterniflora* grows; (b) images were taken under appropriate conditions, such as cloud cover of less than 20%; and (c) images were taken at low tide, so that *S. alterniflora* could be seen growing in the intertidal zone. After the original images were preprocessed by geometric correction, atmospheric correction, image stitching, image striping repair, band synthesis, etc., the remote sensing map of the regional wetland was obtained. On this basis, the visual interpretation of these images was carried out. All raw data were obtained from the Geospatial Data Cloud of the International Scientific Data Service Center (www.gscloud.cn, accessed on 5 June 2021).

In addition, using the above remote sensing images to calculate the vegetation index, i.e., the *NDVI* value, to estimate the vegetation coverage, the calculation formula is:

$$NDVI=\frac{NIR-R}{NIR+R}$$

$$VFC=\frac{NDVI-NDVI_{min}}{NDVI_{max}-NDVI_{min}}$$

where NDVI is the vegetation index, NIR is the reflection value in the near-infrared band, R is the reflection value of the red-light band, VFC is the vegetation coverage, $NDVI_{max}$ is the largest *NDVI* value in the area, and $NDVI_{min}$ is the smallest *NDVI* value in the area.

#### 3.1.2. Terrain Data

**Figure 3:**
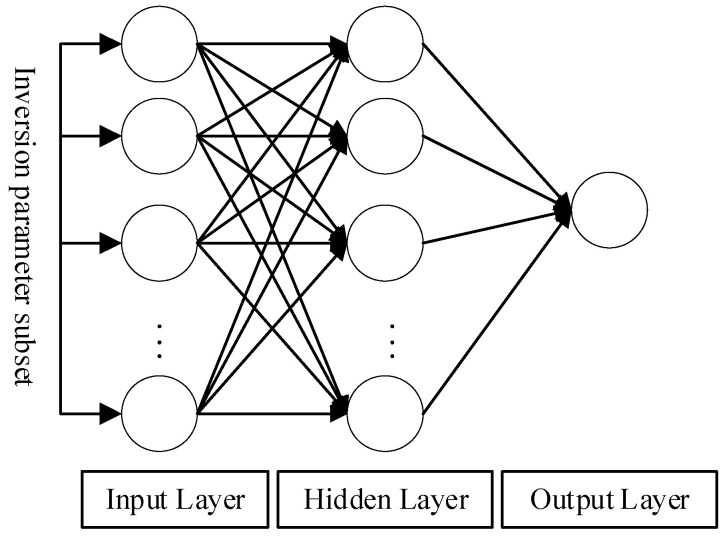
Schematic diagram of the BP neural network structure.

Adopting the field-surveyed elevation data of the 2002 research team and the Lidar elevation data from 2017, we used the BP neural network method to invert the topographic distribution of the region from 1980 to 2017. The core of the BP neural network model is to use an iterative gradient algorithm to correct the weight of the network output and the target by adjusting the error so as to minimize the total error of the network. The BP neural network model includes three parts: network construction, training, and prediction. In this study, 200 collection points were randomly generated from the terrain data, and several types of information, such as vegetation changes, distance from the sea, and vegetation types, were collected. The specific method can be outlined as follows. (1) The principal component score data were used for the input layer of the model, which was randomly divided into 3 subsets, namely the training data set, the verification data set, and the test data set; the proportions were, respectively, 70%, 15%, and 15%. The predicted terrain elevation was the output layer of the model, and the number of neurons in the hidden layer was set and adjusted according to empirical formulas. (2) The forward transmission of information and the backward propagation of errors in the output layer were carried out through the input of a known sample set; the weights and thresholds of the neurons in each layer were continuously adjusted, and they converged along the direction of the most rapid decline in the error function until the error was less than the target. We set the value and then saved the connection weight and threshold information between the layers to establish a prediction model. (3) We used the established prediction model to predict the output of the test sample set (Figure 3). R^2^ (coefficient of determination) was selected to evaluate the simulation accuracy.

### 3.2. Construction of the Material Exchange Function Index

#### 3.2.1. Manning Roughness Coefficient

The roughness of the wetland surface is the main factor affecting material exchange in the intertidal zone. The study used the Manning roughness coefficient to evaluate the surface roughness of the intertidal wetland [33]. The Manning roughness coefficient formula is:

$$n=n_{base}+n_{topo}+n_{vege}$$

where n is the Manning roughness, $n_{base}$ is the soil roughness of the wetland surface, $n_{topo}$ is the topographic roughness of the wetland surface, and $n_{vege}$ is the vegetation roughness of the wetland surface.

According to the distribution characteristics of unused soil, landform, and vegetation, the roughness values of the soil, landform, and vegetation were selected (Table 2). The Yancheng intertidal wetland is a typical muddy tidal wetland, and the wetland surface is covered by sand gravel and broken shells in less than 25% of its area, so the value selected was 0.025.

#### 3.2.2. Material Exchange Function Index

The factors influencing the material exchange function between the land and the sea in the intertidal zone are complicated. A material exchange function evaluation index was constructed to scientifically, objectively, efficiently, and uniformly evaluate the characteristics and the temporal and spatial differences in the material exchange function between the land and sea in the coastal intertidal zone of Yancheng. Since the area was not affected by the invasion of *S. alterniflora* and human activities in 1980, it presents the characteristics of material exchange in the intertidal zone under ideal conditions. For this reason, this study used the 1980 data as the reference wetland, and the material exchange function evaluation index for other years is as follows:

$$H_{i}=\ln\left(\frac{EX_{i}}{EX_{0}}\right)$$

where $H_{i}$ is the evaluation index of the material exchange function of the intertidal zone in the year i; $EX_{i}$ is the material exchange capacity, i=1,2,3,⋯,8, representing 1985, 1990, 1995, ⋯, 2017;$\;\mathrm{and}\;EX_{0}$ is the sea–land material exchange capacity of the Yancheng coastal wetland in 1980.

### 3.3. Methods for Evaluating the Material Exchange Function of the Intertidal Zone

**Figure 4:**
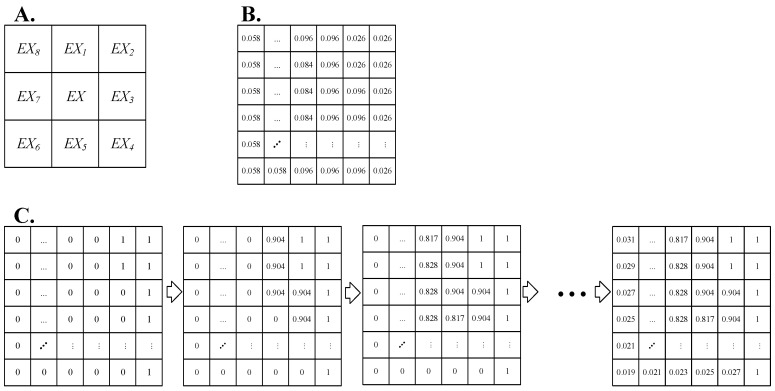
Image calculation process of land–sea exchange capacity. (**A**) Topological relationship and numbering of the surrounding pixels. (**B**) Raster map of Manning’s roughness coefficient. (**C**) Schematic diagram of the calculation process of sea–land exchange capacity.

In order to spatially reflect the heterogeneity of the regional material exchange function, the study area was divided into 100 m × 100 m grid units, with a total of 210 rows × 214 columns. We determined the material exchange capacity of the land and sea in the initial state as 1. The calculation formula for the land–sea exchange capacity of the remaining features is as follows:

$$Ex=Max\left(Ex_{i}\times \left(1-\mu \times n\right)\right)$$

where Ex is the sea–land exchange capability of the pixel; Max() is the maximum value function; and $Ex_{i}$ is the sea–land exchange capability of the surrounding pixels, i=1,2,3,4,5 (the positional relationship of pixels is shown in Figure 4). μ is the adjustment coefficient, for which a value of 0.4 was selected in this paper; n is the Manning roughness coefficient of the pixel.

Using the ArcToolbox > Spatial Analyst Tools > Map Algebra > Raster Calculation module on the ArcGIS platform, the spatial distribution map of the material exchange function index of the typical wetland in Yancheng from 1985 to 2017 was obtained.

## 4. Results

### 4.1. Spatial Differentiation of Wetland Vegetation Types and Coverage

**Figure 5:**
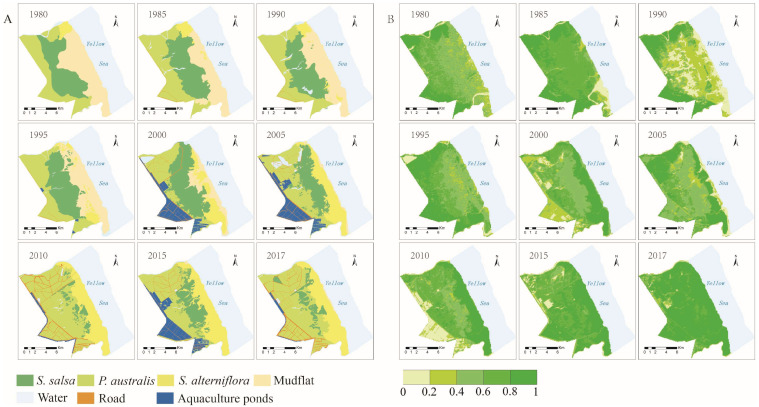
Vegetation coverage distribution map of coastal wetland in Yancheng. (**A**) is the distribution map of coastal wetland vegetation types. (**B**) is the distribution map of coastal wetland vegetation coverage.

**Figure 6:**
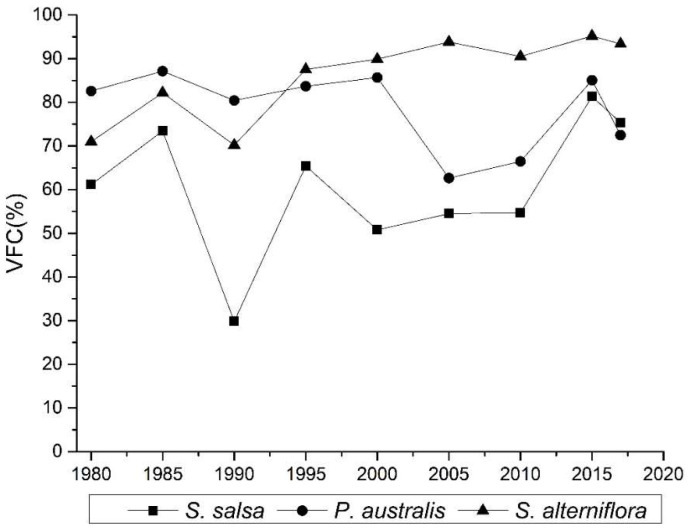
Inter-annual variations in vegetation coverage.

The vegetation coverage in the study area presented spatial heterogeneity (Figure 5). Before 2000, the vegetation coverage increased with the distance from the sea. After 2000, *S. alterniflora* was distributed in the intertidal zone, and its coverage spanned the largest area. The coverage levels of different types of vegetation were not the same. The vegetation coverage of *S. alterniflora* was the highest, with a value of 85.96%, followed by the vegetation coverage of *P. australis*, which had an average coverage of 78.46%; the vegetation coverage of *S. salsa* was the lowest at 60.73%. The vegetation coverage of *S. alterniflora* increased from 70.99% in 1980 to 93.37% in 2017(Figure 6). The vegetation coverage of *P. australis* stabilized at about 78%, although its vegetation coverage in 2005 and 2010 was low at around 60% for both years. The vegetation coverage of *S. salsa* saw a large degree of change. In 1990, its vegetation coverage was 29.81%.

### 4.2. The Spatial Differentiation Characteristics of the Topography of the Intertidal Zone

**Figure 7:**
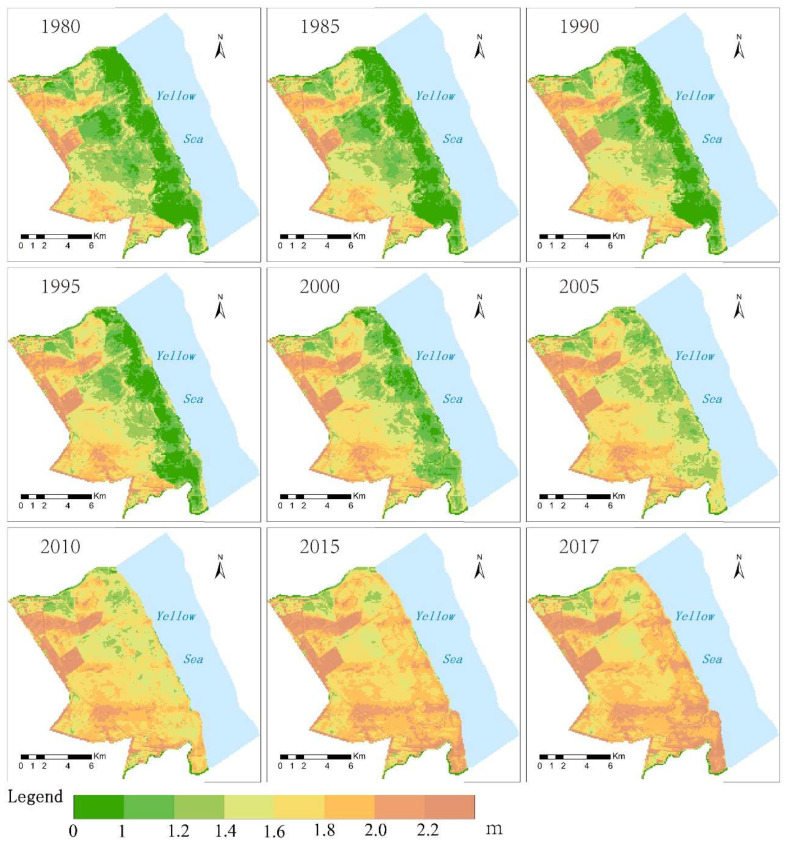
Topographic elevation distribution map of intertidal wetland (1980–2017).

The regional elevation distribution from 1980 to 2017 is shown in Figure 7. After testing, the simulation accuracy was 0.912. It can be seen from the figure that the regional intertidal wetland faces the Yellow Sea in the east, with an average elevation between −0.84 m and 3.04 m, high in the west and low in the east. In terms of spatial changes, the topographic elevation of the *S. alterniflora* distribution area changed greatly from 1980 to 2017. The elevation of the *S. salsa* distribution area increased from 1.42 m in 1980 to 1.72 m in 2017. The *P. australis* beach rose from 1.71 m to 1.85 m, and the terrain height increased by 0.14 m in 37 years. In the distribution area of *S. alterniflora*, the average elevation of the tidal flat in 1980 was less than 1 m, and it was 1.97 m in 2017; in addition, the average elevation increased by 1.24 m. Between 2000 and 2005, the topography of the *S. alterniflora* distribution area rose the fastest, with an increase of 0.34 m in 5 years (Table 3).

### 4.3. Spatial Differentiation of the Surface Roughness of Wetland in the Intertidal Zone

**Figure 8:**
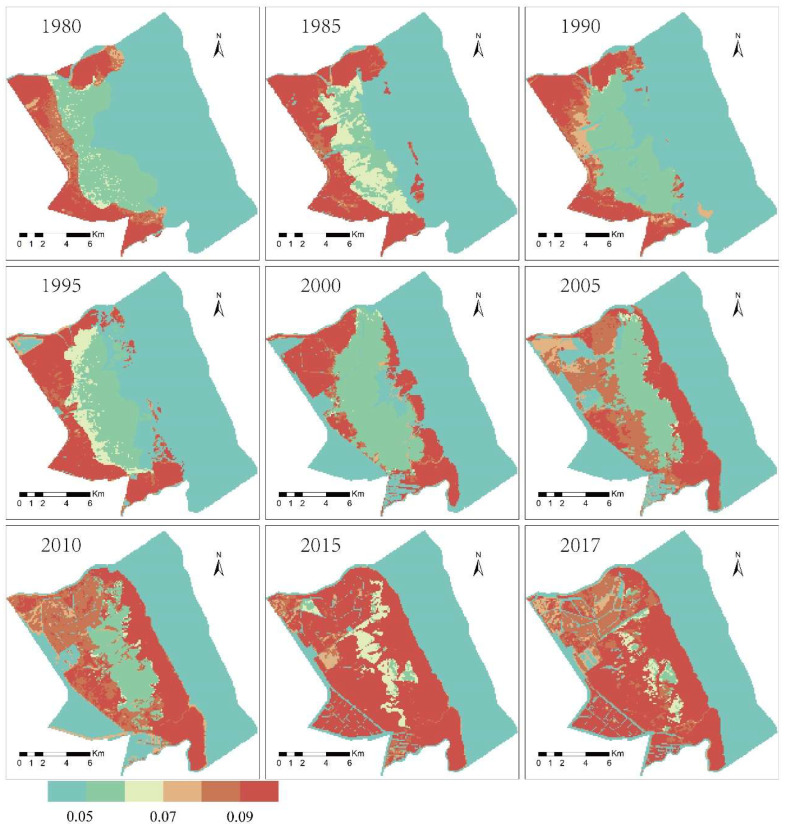
Distribution of Manning’s coefficient in the Yancheng coastal wetland.

**Figure 9:**
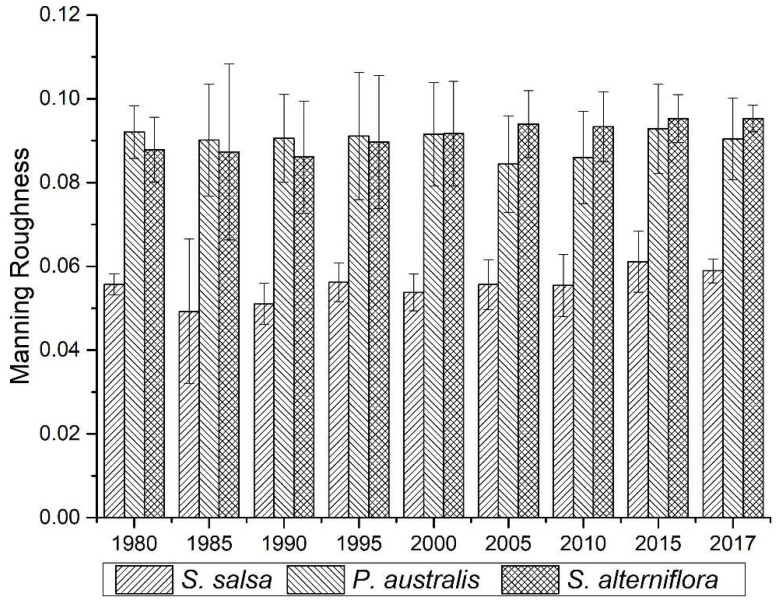
Manning roughness rate changes in different types of vegetation in coastal wetlands.

The spatial differences in the surface roughness of the intertidal wetland were mainly affected by the terrain and vegetation. The topographic distribution map and the vegetation type and coverage distribution map were superimposed and analyzed to form a wetland roughness distribution map of the intertidal zone (Figure 8). From a spatial perspective, before 2010, the surface roughness of the wetlands gradually increased from sea to land. After the invasion of *S. alterniflora*, the surface roughness of the distribution area of *S. alterniflora* gradually increased, which seriously affected the surface roughness distribution of adjacent wetlands. Judging from the changes in the Manning roughness rate of different types of vegetation in coastal wetlands (Figure 9), the average Manning roughness rate of *S. salsa* vegetation was 0.049, and the internal variance was stable. The average Manning roughness rate of *P. australis* was 0.084. From 1980 to 1995, the *P. australis* Manning roughness increased from 0.006 to 0.015, and from 1995 to 2017, the Manning roughness decreased from 0.015 to 0.009. The average Manning roughness rate of *S. alterniflora* was 0.091. From 1985 to 2017, the Manning roughness of *S. alterniflora* decreased year by year, from 0.021 to 0.003.

### 4.4. The Spatial Characteristics and Changes in the Material Exchange Function in the Intertidal Zone

**Figure 10:**
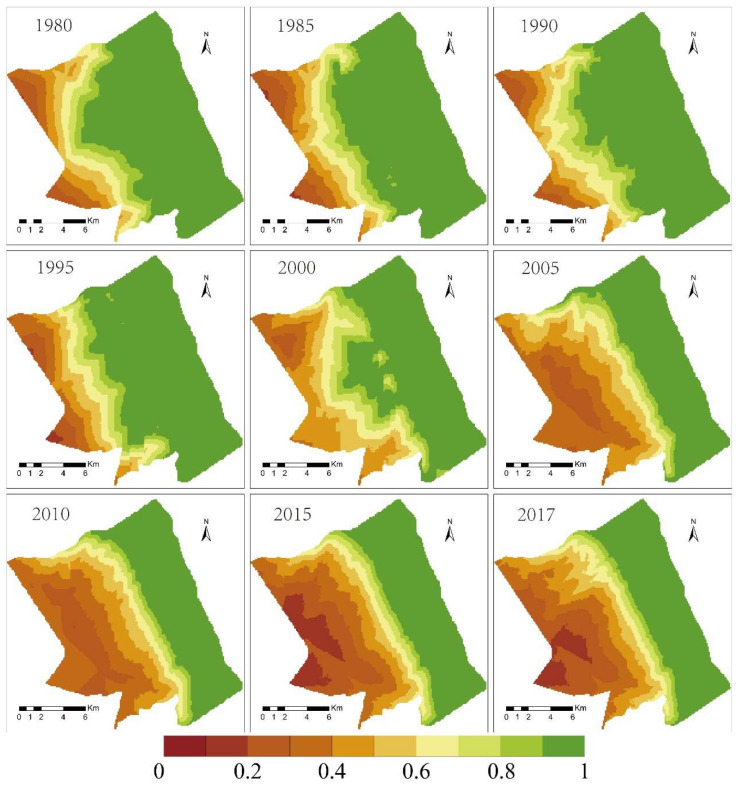
Distribution map of the land–sea material exchange function of the Yancheng coastal wetland.

The material exchange function index and the surface Manning coefficient were closely related, and the material exchange function map from 1980 to 2017 was obtained using the model (Figure 10). The material exchange function between land and sea changed significantly in 37 years. From a spatial point of view, the distribution is high in the east and low in the west, that is, the closer it is to the sea, the stronger the material exchange function between the sea and the land. In terms of the time series, the material exchange capacity in 1980 was 0.85, and in 2017 it was 0.65, which was a decrease of 25%. With time going by, the material exchange capacity changed dynamically, showing an overall downward trend. Specifically, the size of the area with a high material exchange capacity (greater than 0.7) decreased. In 1980, the area with a high material exchange capacity was 39.67 km^2^. In 2017, it was less than 11.34 km^2^; in contrast, the area with a low material exchange capacity (less than 0.4) increased. In 1980, the area with a low material exchange capacity was 17.54 km^2^. By 2017, it was 71.62 km^2^ (Table 4).

## 5. Discussion

### 5.1. Influence of S. alterniflora on Hydrological Processes

The Yancheng intertidal wetland is a typical silty wetland, affected by ocean tides and material deposition [34]. The material exchange function presented a spatial differentiation. It manifested as topographic gradient changes and had the same basic characteristics as the belt-like distribution pattern of vegetation types [35]. The coastal geomorphology process changes the habitat of plants, leading to vegetation succession. The evolution of the coastal landscape pattern is the product of the mutual adaptation of plants and habitats [36,37]. Therefore, changes in plant cover types are the external manifestations of changes in the material exchange function of coastal wetlands [38].

**Figure 11:**
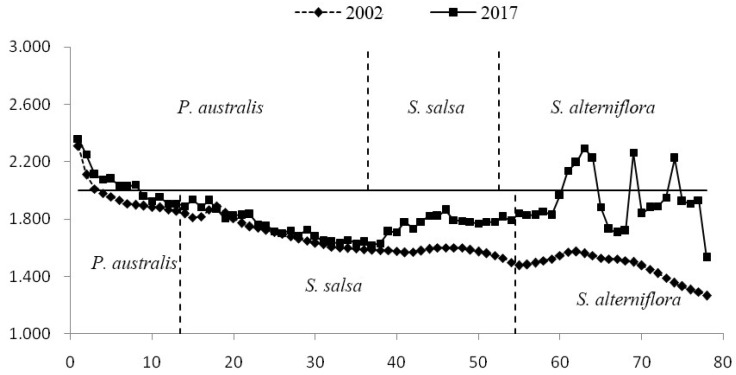
The elevation changes in the cross-section of the coastal wetland in Yancheng.

As time goes by, vegetation types succeed to the sea. However, after the invasion of *S. alterniflora*, especially after 2000, when areas of *S. alterniflora* joined together to form a “biological embankment”, the material exchange function of the intertidal zone changed (Figure 11). This change became more prominent with the expansion of the area of *S. alterniflora*.

The Manning roughness coefficient is determined by vegetation type, vegetation coverage, micro-topography, soil matrix properties, and surface coverage [39,40]. There are three main types of vegetation in coastal wetlands: *S. salsa*, *P. australis*, and *S. alterniflora.* Those contributing to the Manning coefficient were fixed. The variance in the Manning roughness of *S. alterniflora* increased year by year, indicating that the variation in factors such as topography also increased year by year. This was consistent with previous research results.

**Figure 12:**
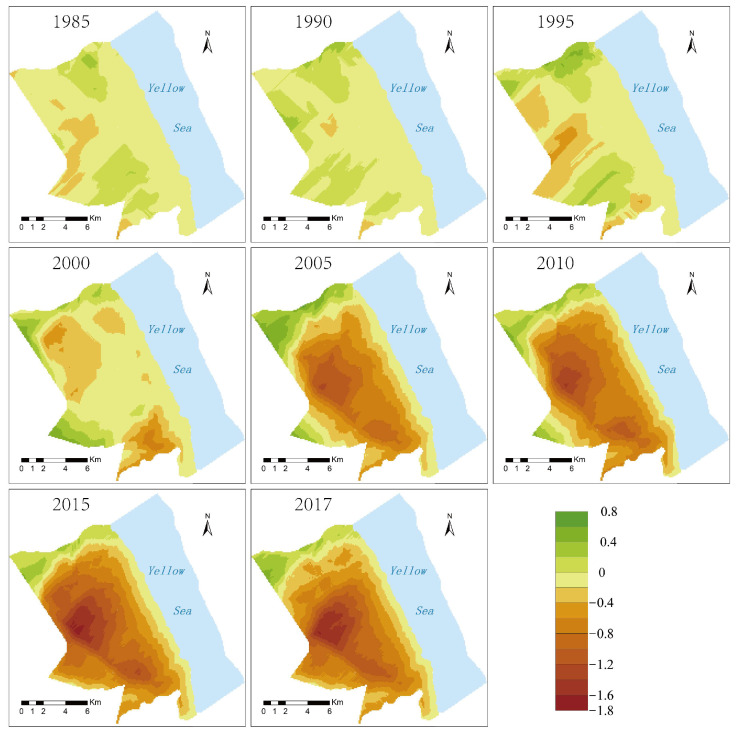
The distribution map of the change in the Yancheng sea–land material exchange function (the larger the value, the stronger the material exchange capacity).

**Figure 13:**
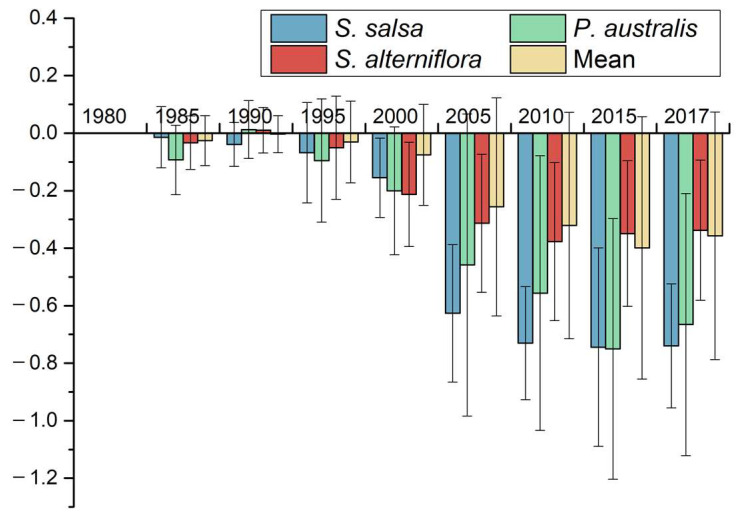
The change in the land–sea exchange function index of different types of vegetation in the coastal wetland of Yancheng.

In this study, based on the material exchange function in 1980, spatial distribution maps of the material exchange function in other years were superimposed and subtracted from 1980 to obtain the spatial change map of the material exchange function in the intertidal zone (Figure 12). Before 2000, there was little change in the material exchange function of the wetland in the intertidal zone of the region. The vegetation types found in the wetland naturally succeeded towards the sea, and the vegetation boundary moved towards the sea. After 2000, the material exchange function of the intertidal zone underwent tremendous changes. The material exchange function evaluation index plummeted from −0.075 in 2000 to −0.2560 in 2005, and then to −0.357 in 2017. From the perspective of wetland vegetation types, the exchange index of the *S. salsa* vegetation zone in 1985 was −0.014, and by 2017, the index was −0.740, representing a significant decrease (Figure 13). This shows that the *S. salsa* ecosystem basically lost its ability to exchange materials with the ocean. The material exchange index of the *P. australis* vegetation zone in 1985 was −0.093, and it was −0.666 in 2017. The change is obvious in this result, too, showing that the *P. australis* ecosystem is basically no longer affected by the exchange of materials between the sea and the land. The change in the material exchange index of the *S. alterniflora* vegetation zone is not obvious. Although the material exchange indices of the three vegetation zones all showed a weakening trend, the degree of weakening of different vegetation zones was different, as follows: *S. salsa* > *P. australis* > *S. alterniflora*. 

### 5.2. Effects of S. alterniflora on the Yancheng Coastal Wetland Ecosystem

The Yancheng coastal wetland’s unique natural factors, such as climate, hydrodynamics, and geomorphic processes, enabled *S. alterniflora* to expand rapidly [41,42]. *S. alterniflora* is in a favorable position in interspecific competition because of its wide ecological range and sexual and asexual reproduction methods; it can grow in most areas of the coastal intertidal zone [43]. With its super expansion ability, *S. alterniflora* expanded, in 2000, into a strip landscape with a north–south connection and a width of several kilometers from east to west. *S. alterniflora* was introduced successfully, and the pioneer community on the ground gradually changed from *S. salsa* to *S. alterniflora*; the succession sequence of light beach → *S. alterniflora* appeared, which led to the disappearance of the succession sequence of light beach → *S. salsa* marsh, forming the succession sequence of *S. salsa* marsh → *S. alterniflora* marsh, which led to the disappearance of *S. salsa* marsh. Nowadays, *S. alterniflora* marsh is basically connected with reed marsh, and *S. alterniflora* has become the dominant community in the region. As a result, the plant community in the study area tends to be of a single type, resulting in a decline in habitat biodiversity; the number of suitable habitats continues to decline [44], and the structure of bird communities will tend to simplify.

The invasion of *S. alterniflora*, with its strong siltation-promoting ability, raised the terrain of the *S. alterniflora* distribution area on the coastline, forming a wide natural embankment [45]. At present, the *S. alterniflora* zone has effectively changed the impact of the original tide on the wetland hydrological process in the intertidal zone, resulting in a reduction in seawater inundation frequency in *S. salsa* distribution area, the destruction of the water–salt balance, and serious ecological degradation. The Yancheng coastal intertidal wetland ecosystem has the basic characteristics of tidal hydrodynamic drive, low topographic gradient, and sheet flow. Under the comprehensive action of these factors, the tidal flooding frequency, scope, time, and other hydrological process cycles, as well as the intertidal zone, present the characteristics of zonal differentiation with respect to the supratidal zone, intertidal zone, and subtidal zone. The input of tidal water brings nutrients necessary for the health of the intertidal wetland system; also in addition, sediment deposition makes the terrain rise, vegetation types continue to evolve to the sea, and various ecosystems develop healthily, orderly, and dynamically. However, since the 1980s, the invasion of *S. alterniflora* has become increasingly prominent. Because *S. alterniflora* plants are tall and dense, they expand rapidly on the light beach that is submerged by the diurnal tide in the intertidal zone, forming a vegetation zone with a width of 1~3 km; this has seriously affected the original tidal process, resulting in disordered changes in the landform and vegetation succession of the intertidal zone and seriously interfering with the healthy and dynamic succession law of the original intertidal zone.

*S. salsa* is a pioneer species in intertidal tidal flat wetlands, and it continues to evolve naturally to the sea under the influence of tidal flooding frequency and sediment deposition. Relevant studies have shown that under the influence of periodic tides, the water, salt, and nutrients of the *S. salsa* ecosystem in the intertidal zone are constantly renewed and exchanged, which not only weakens the water/salt restrictions on plant growth but also provides suitable environmental conditions for benthos; as a result, the ecosystem develops healthily. However, after the invasion of *S. alterniflora*, the frequency and process of tidal flooding into the *S. salsa* distribution area changed. Generally, before the invasion of *S. alterniflora*, *S. salsa* plants were short, and their ability to block the tide was not strong. The tidal flow was mainly sheet flow, flooding the entire *S. salsa* ecosystem; after the invasion of *S. alterniflora*, its dense clumps of tall plants and broad-banded distribution pattern effectively blocked the tidal water flow, weakening the exchange process between the *S. salsa* ecosystem and tidal substances. This changed the water, salt, and nutrition pattern, resulting in the loss of the due elasticity of the *S. salsa* ecosystem, reduced productivity, and obvious ecological degradation [46]. In the reclamation area of the intertidal zone, land use, ditches, roads, and *S. alterniflora* basically blocked the periodic nourishment of tidal water, resulting in the loss of 90% of *S. salsa* wetlands.

### 5.3. Implications of the Land–Sea Material Exchange Function Model

Many coastal native wetlands have been developed and utilized. Human interference and the invasion of alien species have sharply increased the environmental pressure faced by wetland habitats, and coastal wetland ecosystems have undergone significant degradation [47]. Wetland ecological restoration is a costly and complex feat of system engineering that, nevertheless, needs to take the restoration of wetland structure and function in the whole region as its basic goal, with an understanding of the regularity of regional wetland ecological changes and their driving factors. The construction of the sea–land material exchange model can scientifically aid the understanding of the influencing factors that affect the spatial differences in the material exchange function of intertidal wetlands; it can also reveal the spatio-temporal evolution characteristics and laws of the material exchange function of intertidal wetlands.

The introduction of *S. alterniflora* changed the natural succession characteristics and vegetation pattern of the wetland; it also reduced the water volume and material exchange capacity of the wetland. The *S. alterniflora* distribution zone effectively changed the impact of the original tide on the hydrological process of the wetland in the intertidal zone, resulting in a reduction in seawater inundation frequency in the *S. salsa* distribution area, the destruction of the water and salt balance, and serious ecological degradation. In wetland ecological restoration, priority should be given to wetland hydrological restoration.

In our research, we used remote sensing and GIS technology to carry out research on the temporal and spatial changes in the material exchange function of intertidal wetlands, and we scientifically evaluated the impact of *S. alterniflora* invasion on the material exchange function of intertidal wetlands. This method has some limitations.

1. Data source: remote sensing data acquisition is affected by weather factors, and obtaining appropriate images is the key. We easily obtained global elevation data with a spatial resolution of 90 m, but more accuracy requires lidar data to provide high-precision elevation information.
2. Data accuracy: the spatial resolution of Landsat data was 30 m. In order to improve the accuracy of the results, it is necessary to acquire high-resolution satellite images.

## 6. Conclusions

The Jiangsu coastal intertidal wetland is a very sensitive ecosystem affected by ocean tides [48]. The change in the exchange capacity of sea and land directly affects the temporal and spatial succession characteristics and degradation degree of the wetland ecosystem [49]. From the perspective of the material exchange function of the land and sea, this study was conducted by constructing a material exchange function evaluation index and using remote sensing and GIS technology to realize the study of the temporal and spatial changes in the regional material exchange function. The conclusions are as follows:

1. The material exchange function of the wetland ecosystem in the intertidal zone showed a gradual decline from land to sea, and its influencing factors were mainly terrain and vegetation spatial distribution.
2. The invasion of *S. alterniflora* was the main factor in the change in the material exchange function of the wetland in the intertidal zone. The material exchange function of the intertidal zone is an important material guarantee for maintaining the health and natural succession of the wetland ecosystem. Taking the material exchange capacity in 1980 as a reference, the material exchange capacity dropped by 25% in 2017. The size of the area with a high material exchange capacity is decreasing. By 2017, it had been reduced by 71%. In contrast, the size of the area with a low material exchange capacity is increasing; by 2017, it has increased by three times.

## Figures and Tables

**Table 1:** Remote sensing image data source information.

Year	Sensor	Date	Spatial Resolution/m	Cloudiness/%	Data Identification
1980	MSS	28 October 1980	78	0	LM31280371980302AAA03
1985	TM	24 September 1985	30	3	LT51190371985267HAJ00
1990	TM	8 October 1990	30	8	LT51190371990281HAJ00
1995	TM	9 December 1995	30	1	LT51190371995343CLT00
2000	ETM+	9 September 2000	30	53 *	LE71190372000253EDC01
2005	ETM+	26 November 2005	30	6	LE71190372005330EDC00
2010	ETM+	10 December 2010	30	1	LE71190372010344EDC00
2015	OLI	13 October 2015	30	0.04	LC81190372015286LGN01
2017	OLI	5 December 2017	30	0.58	LC81190372017339LGN00

* The cloud cover above the study area was 0, which meets the interpretation requirements.

**Table 2:** Manning roughness coefficient index value.

	Roughness Value			Feature Description
$n_{base}$	0.025			Wetland bare soil background value
	0.030			Covered by gravel and broken shells in more than 25% of area
$n_{topo}$	0.001			A typical flat area without micro-landforms (such as hills) or large-scale landforms (such as narrow channels, tidal channels, mountains, depressions, and ponds)
$n_{vege}$	Vegetation coverage			
	<50%	50~75%	≥75%	
	0.025	0.030	0.035	Short-stemmed soft-leaf area (*S. salsa*)
	0.050	0.060	0.070	High-stemmed and soft-leaf area (*P. australis*, *S. alterniflora*)

**Table 3:** Topographic elevation distribution of vegetation (unit: m).

Year	*S. sals* *a*			*P. australis*			*S. alterniflora*		
	Min	Max	Mean	Min	Max	Mean	Min	Max	Mean
1980	0.33	2.72	1.42	−0.97	4.36	1.71	−0.07	1.59	0.73
1985	0.42	2.23	1.34	−1.00	4.40	1.73	0.19	1.84	0.91
1990	−0.84	2.96	1.52	−0.96	4.43	1.78	0.40	1.87	1.06
1995	0.57	3.04	1.60	−0.61	4.47	1.82	0.53	2.16	1.08
2000	0.71	2.57	1.57	0.20	4.43	1.77	−0.64	2.20	1.16
2005	0.95	2.61	1.54	0.20	4.46	1.78	0.91	2.32	1.40
2010	1.13	2.16	1.63	−0.23	4.50	1.82	−0.43	2.28	1.65
2015	1.19	2.11	1.66	0.88	4.54	1.85	0.30	2.53	1.88
2017	1.26	2.26	1.72	0.51	4.47	1.85	0.71	2.63	1.97
Mean	0.63	2.52	1.56	−0.22	4.45	1.79	0.21	2.16	1.32

**Table 4:** Different vegetation areas with different levels of material exchange function from 1980 to 2017. (Unit: ha).

Years		Material Exchange Function								
		0.1–0.2	0.2–0.3	0.3–0.4	0.4–0.5	0.5–0.6	0.6–0.7	0.7–0.8	0.8–0.9	0.9–1
1980	*S. alterniflora*						0.66	1.41	1.84	1.56
	*S. salsa*				2.68	6.69	7.78	9.38	10.76	9.08
	*P. australis*	0.06	6.88	10.23	8.94	8.39	6	2.76	1.31	0.94
1985	*S. alterniflora*						1.06	1.88	2.05	3.46
	*S. salsa*				0.38	4.04	8.92	9.23	8.15	7.45
	*P. australis*	0.76	9.85	10.13	12.03	9.36	3.92	2.4	1.83	0.81
1990	*S. alterniflora*						0.22	1.07	1.6	3.58
	*S. salsa*				0.08	3.08	8.95	12.94	11.04	10.45
	*P. australis*	0.04	6.05	9.74	10.45	11.61	6.9	2.73	1.29	0.73
1995	*S. alterniflora*						0.5	1.19	1.7	5.93
	*S. salsa*				1.16	7.28	8.9	10.07	9.62	11.39
	*P. australis*	1.22	7.91	13.77	10.58	5.79	4.06	2.37	1.37	0.42
2000	*S. alterniflora*				0.47	1.69	2.12	3.61	6.37	7.79
	*S. salsa*				0.73	6.4	12.85	14.3	10.56	8.53
	*P. australis*		3.09	8.14	10.17	8.03	2.41	0.64		
2005	*S. alterniflora*			0.62	2.01	4.04	5.55	8.02	7.66	5.93
	*S. salsa*		1.3	3.69	12.21	9.88	4.72	1.77	1.19	0.15
	*P. australis*		10.45	17.78	10.84	4.72	3.01	1.79	0.76	0.15
2010	*S. alterniflora*		0.01	1.65	4.85	6.06	7.04	9.19	8.12	5.66
	*S. salsa*		0.35	7.36	10.55	6.22	2.45	0.77	0.21	
	*P. australis*	0.02	18.85	22.06	8.09	4.02	2.63	0.91	0.08	0.11
2015	*S. alterniflora*			1.01	3.08	6.13	6.85	9.47	7.94	5.27
	*S. salsa*		1.47	5.76	4.19	1.77	1.98	0.42	0.25	0.01
	*P. australis*	15.18	27.72	20.27	13.04	4.76	2.19	0.69	0.05	
2017	*S. alterniflora*			0.63	3.19	5.75	6.94	9.17	8.07	5.22
	*S. salsa*		0.11	2.51	3.6	2.33	1.2	0.28		
	*P. australis*	9.6	26	22.23	16.92	7.61	3.91	1.08	0.21	0.01

## Data Availability

The datasets generated and/or analyzed during the current study are available from the corresponding author on reasonable request.
